# A New Surrogating Algorithm by the Complex Graph Fourier Transform (CGFT)

**DOI:** 10.3390/e21080759

**Published:** 2019-08-04

**Authors:** Jordi Belda, Luis Vergara, Gonzalo Safont, Addisson Salazar, Zuzanna Parcheta

**Affiliations:** 1 Institute of Telecommunications and Multimedia Applications, Universitat Politècnica de València, 46022 València, Spain; 2 Sciling SL, C/Camí a La Mar 75, 46022 València, Spain

**Keywords:** surrogates, graph Fourier transform, Hermitian Laplacian matrix

## Abstract

The essential step of surrogating algorithms is phase randomizing the Fourier transform while preserving the original spectrum amplitude before computing the inverse Fourier transform. In this paper, we propose a new method which considers the graph Fourier transform. In this manner, much more flexibility is gained to define properties of the original graph signal which are to be preserved in the surrogates. The complex case is considered to allow unconstrained phase randomization in the transformed domain, hence we define a Hermitian Laplacian matrix that models the graph topology, whose eigenvectors form the basis of a complex graph Fourier transform. We have shown that the Hermitian Laplacian matrix may have negative eigenvalues. We also show in the paper that preserving the graph spectrum amplitude implies several invariances that can be controlled by the selected Hermitian Laplacian matrix. The interest of surrogating graph signals has been illustrated in the context of scarcity of instances in classifier training.

## 1. Introduction

### 1.1. Statement of the Problem and Related Works

Starting from an available original signal, synthetic signals can be generated which are surrogates of it. Essentially, surrogate means that the (Fourier) spectrum amplitude (we will use the term amplitude to define the magnitude or modulus of a complex number or a real number) of the original signal is retained while the phase is randomized. Surrogating methods have been considered in a variety of applications [1,2,3,4,5,6,7], where the synthetic signals are used to obtain a non-parametric estimate of the distribution of some statistic considered in a given test of hypotheses. In spite of its attractive approach, existing surrogating methods are too constrained due to the use of the Fourier transform (FT)—Both the original signal and its surrogates share the same autocorrelation function. This implies that pairwise interrelations are assumed to be the same for pairs of samples equally separated. While this can be appropriate in some cases, much more flexibility would be gained if specific pairwise relations between samples could be imposed in the surrogates. Thus, for example, we could enhance short-term properties by prioritizing interrelations between neighbor samples. We could also introduce application domain interrelation constraints given by an expert that must agree with the surrogates. In fact, we will show that FT methods are particular cases of the newly proposed method.

In this paper, we propose a new method for generating surrogates which exploit the concept of graph signal processing [8,9,10]. Graph signal processing (GSP) is an emerging area which extends the classical concept of signals defined on a uniform 1-D (time signal) or 2-D (images) grid, to more general grids. Thus it allows the ability to model interrelations between samples in a more general way and benefits from consolidated concepts and methods emanating from signal processing and related areas. Notice that a graph signal may represent any arbitrary domain: Time samples, image pixels, a vector of features extracted from some preprocessing, the outputs of a sensor network, and so on.

In a previous related work [11], the authors propose a sign randomization of the graph Fourier transform (GFT). The GFT represents the graph signal in a domain expanded by the eigenvectors of the graph Laplacian matrix (other GFT are possible like the one proposed in [12], which considers the generalized eigenvectors of the adjacency matrix. This may lead to further extensions of the work presented here) [8,13]. The algorithm is applied to generate surrogates of EEG signals defined in the space domain, which preserve the smoothness of the original signals in the defined graph. The authors demonstrate the interest of the method in the detection of the effects of behavioral tasks in electroencephalography signals. The good results of this novel approach suggest continuing to working on it.

### 1.2. New Contributions and Paper Organization

So, in this paper, we go deeper into the concept in several aspects. First, the complex case is considered. Notice that the GFT of a real graph signal is also real, so phase randomization is actually limited to be a sign randomization as the authors do in [11]. However, working with complex signals allows unconstrained phase randomization. But a complex signal should be expressed as a linear combination of complex eigenvectors. Thus we have defined a complex Hermitian Laplacian matrix (Section 2.1), while most of the previous work on GSP assumes real Laplacian matrices. We derive properties of the Hermitian Laplacian, in particular, a generalization of the smoothness [8,14,15,16] relative to the conjugate complex weights. Then, we generalize the GFT to the complex case (Section 2.2) defining a Complex GFT (CGFT).

Besides, with the aim of gaining understanding and motivation of the proposed surrogating algorithm, we have made an analysis of what properties of the original signals are preserved in the surrogates. This is presented in Section 3 through the concept of graph spectrum amplitude (GSA) invariants. In particular, we demonstrate that the smoothness, the precision matrix [17,18], and the covariance matrix of graph wide-sense stationary (GWSS) stochastic signals, as recently defined in [19,20] are GSA invariants.

In Section 4.1 we present the new surrogating algorithm. First, existing algorithms based on the FT are briefly reviewed. We also show that these algorithms are equivalent to a particular definition of graph connectivity. The issue of how to select a Hermitian Laplacian matrix is considered in Section 4.2.

Finally, Section 5 presents some experiments to assess the improvements of the new surrogating method based on the CGFT in comparison with the one based on the FT. The application target is the training of a classifier with a scarcity of data. This may be considered as a generalization of the classical hypothesis testing problem where surrogates are routinely considered. Thus Section 4.1 is devoted to present experiments of hand gestures recognition. This is a difficult classification problem due to the large number of classes, large, and variant number of features.

### 1.3. Notation

Lower or upper case italics denote a scalar; lower case bold letters denote vectors with $v_{n}$ denoting the *n-*th element of v; upper case bold letters denote matrix with V(n,m) or $v_{nm}$ denoting the (*m*,*n*)-th element of V and $v_{n}$ the *n*-th column; ${\left(\cdot \right)}^{*},{\left(\cdot \right)}^{T},\;{\left(\cdot \right)}^{H}$ denote conjugate, transpose, and conjugate transpose; Re[⋅]≡[⋅]R, Im[⋅]≡[⋅]I denote real and imaginary parts; | ⋅ |,  ‖ ⋅ ‖  denote modulus and Euclidian norm; ∡v denotes phase of the complex number v; detV denotes determinant; $\hat{v}$ denotes a surrogate of v; E[⋅] denotes expectation; $C_{vv}$, $Q_{vv}=C_{vv}^{-1}$ denote the covariance and the precision matrices of the random vector v; $v^{\left(i\right)},\;V^{\left(i\right)}$ denote vector or matrix values corresponding to *i*-th iteration of an iterative algorithm. We will use j to indicate the complex number verifying $j^{2}=-1$.

Finally, a list of acronyms is included in Appendix C, to facilitate tracking.

## 2. The Hermitian Laplacian Matrix

### 2.1. Definition and Properties

There are not many precedents of using complex Laplacian matrices. In [21] a Hermitian Laplacian matrix is proposed to include some possible oriented edges, but this leads to a very limited form of Hermitian Laplacian matrices—Its off-diagonal elements are 1 for non-oriented edges and ±j for the oriented edge. A more general Hermitian Laplacian matrix is proposed in [22] by adding a phase term to the off-diagonal elements of a real Laplacian matrix. Then some permutation invariants are found in the pattern recognition context. This extension implies that the real part of the corresponding adjacency matrix is not guaranteed to have positive entries. Here we propose a different Hermitian Laplacian matrix by adding an imaginary part to the (positive) off-diagonal elements of the adjacency matrix, while the *n*-th element of the diagonal matrix **D** is kept as the sum of the real parts of the edges incident to node *n*. So, let us consider the weighted graph G{V,E,W}, where V represents the set of *N* vertices or nodes of the graph, E represents the set of edges connecting the nodes, and W is the adjacency matrix. The generic element W(n,m) is the weight corresponding to the edge connecting node *m* to node *n*. It is rather common in the literature to assume W to be a real and symmetric matrix with non-negative elements W(n,m)≥0     ∀n,m. While this seems reasonable for real graph signals, it seems too constrained for complex graph signals. Thus we will consider that W is, in general, a Hermitian matrix $W=W^{H},\;W\left(n,m\right)=W^{*}\left(m,n\right)\;\forall n,m$. We will consider that the real part is semi-positive Re[W(n,m)]≥0     ∀n,m. This allows including the real case as a special case. Notice that in the real case all the edges are undirected because W(n,m)=W(m,n)     ∀n,m. However, in the complex case, the imaginary part of the complex weights changes the sign depending on the direction Im[W(n,m)]=−Im[W(m,n)], so these edges are directed. This situation is sometimes known as mixed graphs [21].

We may define a Hermitian Laplacian matrix in the form L=D−W where D is a diagonal matrix having the generic element D(n,n)=∑m=1NRe[W(n,m)]. This is an obvious generalization of the real case where $D\left(n,n\right)=\sum_{m=1}^{N}W\left(n,m\right)$ and D is termed the degree matrix. The foregoing definition of the Hermitian Laplacian matrix is appropriate for extending the concept of smoothness to complex graph signals. Let us define the graph signal $s={\left[s_{1},\ldots ,s_{N}\right]}^{T}\in \;C^{N}$, where the value $s_{n}$ is associated with the *n*-th node of the graph, for n=1…N. Notice that s may correspond to any arbitrary domain.

The graph smoothness can be defined as a simple generalization of the real case

(1) $$S\left(s\right)=s^{H}Ls$$

We show in Appendix A that

(2) S(s)=sHLs=∑n=1N−1∑m>nNwnmR|sn−sm|2+∑n=1N−1∑m>nN2wnmI Im[sn∗sm]

where wnmR≡ Re[W(n,m)] and wnmI≡Im[W(n,m)]. Notice that if the Laplacian matrix and/or the graph signal are real, the second term in Equation (2) vanishes, and the smoothness becomes the classical weighted measure of the (squared) signal differences between every two samples. But in the most general case where both the Laplacian matrix and the graph signal may be complex, a new term appears. The latter can be expressed in the form:

(3) ∑n=1N−1∑m>nN2wnmI Im[sn∗sm]=∑n=1N−1∑m>nN2wnmI|sn| |sm|sin(∡sm−∡sn)

where $s_{n}=\left|s_{n}\right|\exp\left(j∡s_{n}\right)$. Hence, by using the defined Hermitian Laplace matrix, we are incorporating into the smoothness information about the phase difference of the value assigned to node *m* with respect to node *n*. Let us illustrate it with a simple example—A line graph, i.e., a graph in which node *n* is connected only with node *n* + 1 and node *n* − 1, except node 1 which is connected only with node 2, and node *N* which is connected only with node *N* − 1. Let us also assume that the weights are constants, and let us define $s_{r}={\left[s_{N},\ldots ,s_{1}\right]}^{T}$, a reversed version of s. It is straightforward to show that the first term of Equation (1) is the same for both the smoothness of s and $s_{r}$, while the second term changes the sign. Thus the smoothness depends on graph signal amplitude variability over the graph (as it happens in the real case) and on the sense of rotation when moving in the complex plane from one node to the other. This second term can be positive or negative, so the smoothness could eventually be negative for some graph signals. This would imply that the corresponding Hermitian Laplacian matrix could not be a positive semidefinite as a real Laplacian matrix is. This can be demonstrated using Sylvester’s criterion [23] which states that a necessary and sufficient condition for a Hermitian matrix to be positive definite is that all its leading principal minors (determinants of the leading submatrices) are positive. Let us call $L_{2}$ the (2 × 2) leading submatrix of L.

(4) $$\begin{array}{l} L_{2}=\left(\begin{array}{cc} w_{12}^{R}+\sum_{m>2}^{}w_{1m}^{R} & -w_{12}^{R}-jw_{12}^{I}\\ -w_{21}^{R}-jw_{21}^{I} & w_{21}^{R}+\sum_{m>2}^{}w_{2m}^{R} \end{array}\right)\;=\left(\begin{array}{cc} w_{12}^{R}+\sum_{m>2}^{}w_{1m}^{R} & -w_{12}^{R}-jw_{12}^{I}\\ -w_{12}^{R}+jw_{12}^{I} & w_{12}^{R}+\sum_{m>2}^{}w_{2m}^{R} \end{array}\right)\;\\ \det L_{2}=\left[w_{12}^{R}\sum_{m>2}^{}\left(w_{1m}^{R}+w_{2m}^{R}\right)+\sum_{m>2}^{}w_{1m}^{R}\sum_{m>2}^{}w_{2m}^{R}\right]-{\left(w_{12}^{I}\right)}^{2}\; \end{array}$$

We see that depending on the selection of the weights corresponding to the nodes 1 and 2, the leading principal minor of order 2 could be non-positive. The result, Equation (4), could be applied to any pair of nodes as reordering the nodes leads to a new Hermitian Laplacian matrix which is permutation similar to the original one [24], and the positive or non-positive semidefinite condition remains the same.

In consequence, given that the contributions to the second term in Equation (2) may be positive or negative, one may wonder about the actual meaning of increasing or decreasing the smoothness in the complex case. In the real case, this has an intuitive answer, as the smoothness is a measure of graph signal variability on the graph. However, in the complex case, the variability measure of the first term can eventually be compensated by negative contributions of the second term. Hence, to give meaning (even intuitive) to Equation (2) we must consider particular graph topologies (as we did in the above example of a line graph) or define some constraints to the graph signals whose smoothness is being compared. Thus, for example, Equation (2) is a measure of graph signal variability on a given graph, for the graph signal subset satisfying $sign\left[w_{nm}^{I}\right]=sign\left[sin\left(∡s_{m}-∡s_{n}\right)\right]$ so that all the contributions of the second term in Equation (2) are positive. By doing so we guarantee that both the distances and the phase differences make positive contributions to the smoothness, and that zero smoothness is obtained only when $s_{n}=s_{m}\;\forall nm\;$, as it is in the real case.

### 2.2. The Complex Graph Fourier Transform (CGFT)

Any Hermitian matrix can be diagonalized by a unitary matrix, then

(5) $$L=U\Lambda U^{H}=\sum_{n=1}^{N}\lambda_{n}u_{n}u_{n}^{H}$$

where $\lambda_{n}\;n=1\ldots N$ are the eigenvalues of L (Λ a diagonal matrix, $\Lambda \left(n,n\right)=\lambda_{n}$) and $u_{n}\;n=1\ldots N$ (columns of U) are the corresponding orthonormal eigenvectors, $U^{H}=U^{-1}$ so U is unitary. The eigenvalues are real due to the Hermitian property. The GFT is defined for the real case as the projection of the graph signal on the vector space expanded by a basis formed by the eigenvectors of the real Laplacian matrix. Then the notion of frequency is assimilated to the eigenvalue associated with every eigenvector in much the same manner as every complex exponential is defined by a specific frequency when using the FT. We may extend this concept to the complex case by simply considering the eigenvectors of the Hermitian Laplacian, hence defining the direct and inverse CGFT as

(6) $$r\;=CGFT\left(s\right)=U^{H}s\;,\;s=CGFT^{-1}\left(r\right)=Ur\;$$

In the real case, the Laplacian matrix is positive semidefinite, then $\lambda_{n}\;\geq 0\;\forall n$, in fact, the minimum eigenvalue is 0 [25]. However, we have seen that in the complex case the Hermitian matrix **L** could not be positive semidefinite, so at least one eigenvalue will be negative. Notice that the different modes (eigenvectors) of a Hermitian matrix can be ordered according to increasing smoothness because $u_{n}^{H}Lu_{n}=\lambda_{n}$ and that for any vector s $\lambda_{\min}\leq \frac{s^{H}Ls}{s^{H}s}\leq \lambda_{\max}$. Hence we can still assimilate the concept of frequency to the eigenvalue, i.e., CGFT^−1^ in Equation (6) expresses the graph signal as a linear combination of modes having increasing smoothness (from $\lambda_{\min}$ to $\lambda_{\max}$). However notice that, as explained at the end of Section 2.1, it is difficult to get a general interpretation of the meaning of smoothness. Thus the appearance of negative eigenvalues suggests that the sense of rotation when moving in the complex plane from one node to the other can be relevant for a complete characterization of a graph signal by the CGFT. This has been verified in the particular line graph example of the previous section. Another simple numerical example follows. Let us consider a graph of two nodes, where $w_{12}=1+j=w_{21}^{*}$. If we compute the two eigenvalues and two eigenvectors of the corresponding 2 × 2 Hermitian Laplacian we obtain $\lambda_{1}=-0.41$ and $\lambda_{2}=2.41$, $u_{1}={\left[0.5+0.5j,\;0.707\right]}^{T}$, and $u_{2}={\left[-0.5-0.5j,\;0.707\right]}^{T}$. We see that a negative eigenvalue appears and that in the first eigenvector there is a clockwise rotation when moving in the complex plane from its first element to the second. However, in the second eigenvector, the rotation is counterclockwise. Similarly, we have shown at the end of Section 2.1 that the newly defined smoothness may keep the classical meaning of variability measure by imposing some constraints to the graph signals to be compared. Now, this measure incorporates distances as well as phase differences, which is consistent with the complex domain. Finally, notice that FT can be interpreted as a particular case of a CGFT (as it is of GFT) where the associated Hermitian Laplacian is a circulant matrix (see below, end of Section 4.1).

## 3. Graph Spectrum Amplitude Invariants

The key element of the surrogating algorithms is to keep the GSA of the original graph signal while the graph spectrum phases are randomized. So let us find GSA invariants before presenting the detailed algorithms in the next section. This helps to motivate the graph methods and to gain insights into the implications of surrogating graph signals. GSA invariants are those functions or statistics of the original graph signal which do not change if the GSA does not change. Given a signal s, the set of signals having the same GSA as s is

(7) $$\hat{s}={\left(U^{H}\right)}^{-1}\Phi U^{H}s=U\Phi U^{H}s$$

where Φ is the set of NxN diagonal matrices whose diagonal coefficients are unimodular, i.e., $\Phi \left(n,n\right)=\exp\left(j\phi_{n}\right)\;n=1\ldots \;N$. Notice that the spectral amplitudes of the transformed vector are retained, but a phase $\phi_{n}$ is added to the *n-*th graph spectrum phase, before the inverse transformation. Let us define the unitary matrix $P^{H}=U\Phi U^{H}=P^{-1}$, from Equation (7) we can write

(8) $$\hat{s}{\hat{s}}^{H}=U\Phi U^{H}ss^{H}U\Phi^{H}U^{H}=P^{-1}\;ss^{H}\;P$$

Then the sample correlation matrices of the original graph signal and its surrogates are unitarily similar [26]. Specht’s criterion [27] establishes a necessary and sufficient condition for the unitary similarity of two matrices in terms of some traces invariance. The application of this criterion to the sample correlation matrices leads to

(9) $$trace\left({\left(\hat{s}{\hat{s}}^{H}\right)}^{k}\right)=trace\left({\left(ss^{H}\right)}^{k}\right),$$

for any integer *k*. Equation (9) defines invariants functions involving polynomials of the elements of s and $\hat{s}$. In particular, for k=1 it implies that the energy of the graph signal is a GSA invariant

(10) $$\sum_{n=1}^{N}{\left|{\hat{s}}_{n}\right|}^{2}=\sum_{n=1}^{N}{\left|s_{n}\right|}^{2},$$

and for k=2 we can see that the Frobenius norm of the sample correlation matrix is also a GSA invariant

(11) $$\sum_{n=1}^{N}\sum_{m=1}^{N}{\left|{\hat{s}}_{n}{\hat{s}}_{m}^{*}\right|}^{2}=\sum_{n=1}^{N}\sum_{m=1}^{N}{\left|s_{n}s_{m}^{*}\right|}^{2}.$$

Notice that, so far, the value of the GSA invariant is the same for every possible Hermitian Laplacian matrix, i.e., they are implicit in the preservation of the GSA. Let us consider invariants which depend on the Hermitian Laplacian. First, notice that s and $\hat{s}$ are equally smooth on graph G{V,E,W},

(12) $$\begin{array}{ll} S\left(\hat{s}\right)={\hat{s}}^{H}L\hat{s} & =\;s^{H}U\Phi^{H}U^{H}LU\Phi U^{H}s\\ & =s^{H}U\Phi^{H}U^{H}U\Lambda U^{H}U\Phi U^{H}s\\ & =s^{H}Ls\;=\;S\left(s\right)\;, \end{array}$$

so the smoothness on the graph defined by the selected Hermitian Laplacian is a GSA invariant.

Let us now adopt a stochastic perspective. We assume that s is a zero mean random vector having a known precision matrix $Q_{ss}=C_{ss}^{-1}$, where $C_{ss}=E\left[ss^{H}\right]$ is the covariance matrix. Taking expectations and inverting in Equation (8) we conclude that the precision matrices are also unitarily similar

(13) $$Q_{\hat{s}\hat{s}}=P^{-1}\;Q_{ss}\;P.$$

The elements of $Q_{ss}$ properly normalized are the partial correlations [28,29,30,31] defining linear conditional dependences between every two elements of s. This motivates the extended use of $Q_{ss}$ as the Laplacian matrix. So, let us assume that we select $L=Q_{ss}$, then, considering Equation (5), and the definition of P, we get:

(14) $$Q_{\hat{s}\hat{s}}=U\Phi U^{H}Q_{ss}U\Phi^{H}U^{H}\;=U\Phi U^{H}U\Lambda U^{H}U\Phi^{H}U^{H}=Q_{ss}.$$

Hence the precision matrix is invariant to the GSA when used as the Hermitian Laplacian. Notice that the interpretation of the smoothness in Equation (2) is still valid in this case. This is demonstrated in [17] for the real Laplacian and can be straightforwardly extended to the Hermitian Laplacian as the complex component only affects the nondiagonal elements.

Finally, some recent works [19,20] have proposed equivalent extensions of the concept of stationarity to graph signals by considering definitions of graph signal translation. Then, in [19,20] the authors demonstrate that a stochastic graph signal is GWSS if the covariance matrix is jointly diagonalizable with the Laplacian matrix. So let us assume that the pairwise interrelations are defined such that s is a realization of a (zero mean) random graph signal which is GWSS with respect to the Laplacian used to generate the surrogates, i.e., that the graph signal is invariant to some defined operator of translation, then $C_{ss}=U\Gamma_{ss}U^{H}$, where $\Gamma_{ss}$ is a diagonal matrix, hence

(15) $$C_{\hat{s}\hat{s}}=U\Phi U^{H}U\Gamma_{ss}U^{H}U\Phi^{H}U^{H}\;=C_{ss}.$$

Therefore, the covariance matrix is a GSA invariant for GWSS signals, which is consistent with the well-known invariance of the autocovariance function with respect to the power spectral density of (conventional) WSS signals. Notice that the preserved covariance matrix is not necessarily Toeplitz as it is implicitly assumed when preserving the autocorrelation function with classical surrogating methods. Table 1 summarizes the GSA invariants demonstrated in this section.

## 4. Surrogating Algorithms

### 4.1. Iterative Surrogate Graph Signal Algorithm

One of the first proposals of a surrogating algorithm was the amplitude adjusted Fourier transform (AAFT), [32]. The FT of the original (real) signal is computed, then the samples obtained after spectrum phase randomization followed by inverse Fourier transform are corrected to comply with the empirical distribution of the original samples. Random phases are obtained by sampling a uniform distribution between −π and π, constrained to keep the antisymmetry property of the spectral phases of real signals. Corrections consist of replacing every sample of the surrogate so obtained, by the value of the original sample having the same rank order, thus recovering the empirical distribution of the original signals. Hence, the surrogate is a shuffled version of the original signal.

An improved version which iteratively adjusts the spectrum amplitude and corrects the sample empirical distribution was proposed—Iterative AAFT (IAAFT) [1,33]. Minor improvements or variations of the AAFT have been proposed in [34,35,36]. More recently, extensions to the multivariate case have been considered where the added problem is to keep the correlation among the different surrogate channels [37,38,39,40]. 

So far, these surrogating algorithms have been applied only to real signals in the time domain. We present in this section an iterative algorithm for surrogating complex graph signals by means of the CGFT (ICGFT). Everything starts with the original graph signal $s={\left[s_{1},\ldots ,s_{N}\right]}^{T}\in \;C^{N}$. We compute an initial uncorrected surrogate ${\hat{s}}^{\left(0\right)}$ by randomizing the phases of the CGFT of s (Equation (16a)) while the GSA is maintained. Random phases are obtained by sampling a uniform distribution between −π and π (no antisymmetry constraint is imposed as complex signals are considered). Then ${\hat{s}}^{\left(0\right)}$ is corrected. This correction can be made to match the empirical distribution of the original graph signal [1,33], but other corrections could be better suited to the specific application context (see Section 5), so we will represent the correction by a generic function C(⋅). Thus we get the initial corrected surrogate $s^{\left(0\right)}$ (Equation (16b)) as

(16a) $${\hat{s}}^{\left(0\right)}=U\Phi U^{H}s,$$

(16b) $$s^{\left(0\right)}=C\left({\hat{s}}^{\left(0\right)}\right).$$

Notice that in general correction C(⋅) will modify the GSA, so we proceed iteratively, as in IAAFT, computing the CGFT of the corrected surrogate, recovering the original GSA and generating a new uncorrected surrogate ${\hat{s}}^{\left(i\right)}$ Equation (17a). The latter is again corrected to match the empirical distribution of the original graph signal Equation (17b), and thus successively:

(17a) $${\hat{s}}^{\left(i\right)}=UA^{\left(i-1\right)}U^{H}s^{\left(i-1\right)},$$

(17b) $$s^{\left(i\right)}=C\left({\hat{s}}^{\left(i\right)}\right)\;i=1\ldots I.$$

Defining $r^{\left(i-1\right)}=U^{H}s^{\left(i-1\right)}$ then $A^{\left(i-1\right)}$ is a diagonal matrix having elements $A^{\left(i-1\right)}\left(n,n\right)=\left|r_{n}\right|/\left|r_{n}^{\left(i-1\right)}\right|$. This iterates until convergence, i.e., until no corrections are required. Algorithm 1 below is a pseudocode description of ICGFT.

**Algorithm 1:** Iterative surrogating complex graph signals by means of the **CGFT** (ICGFT).1: **Input:** Graph signal s, Hermitian Laplacian L, number of surrogates *K*, maximum number of iterations *I*, convergence threshold ε
2: **Compute eigenvector matrix**
U
3: **for**
*k* = 1, 2, …, *K*
**do:** 4: Generate graph spectrum random phases $\Phi_{k}$ 5: Compute initial surrogate, Equation (16a,b) $s_{k}^{\left(0\right)}=C\left(U\Phi_{k}U^{H}s\right)$ 6: **for**
*i* = 1, 2, …, *I*
**do:** 7: Compute surrogate, Equation (17a,b) $s^{\left(i\right)}=C\left(UA^{\left(i-1\right)}U^{H}s^{\left(i-1\right)}\right)$ where $A^{\left(i-1\right)}=diag\left(\left|r_{n}\right|/\left|r_{n}^{\left(i-1\right)}\right|\right)$
8: If $\left\|s^{\left(i\right)}-s^{\left(i-1\right)}\right\|<\varepsilon \;\mathrm{then}\;s_{k}=s^{\left(i\right)}$ and go to 11 9: **end for** 10: $s_{k}=s^{\left(I\right)}$
11: **end for**
12: **Output:**
$s_{1}\;\ldots .\;s_{K}\;$

Unfortunately, there is not a clear understanding or theoretical analysis of convergence in all the different surrogating algorithms proposed so far. However, it has been experimentally verified that convergence is reached after a few iterations in most cases. Notice that measuring how close $s^{\left(i-1\right)}$ is to $s^{\left(i\right)}$ in line 8 of Algorithm 1 (i.e., fitting the threshold ε) is arbitrary, with no control of the computational time required to generate surrogates. That is why a maximum number *I* of iterations is allowed. The value ε may be experimentally adjusted in every particular application. In any case, notice that, ultimately, the performance indicators of the underlying application will assess the validity of the generated surrogates rather than a specific criterion to fit ε. Thus, for example, in all the experiments of Section 5, we have finally set a constant number of 10 iterations, i.e., ε=0     I=10, after verifying that there was not a substantial improvement in the performance indicator (probability of error) with an (experimentally) optimized selection of ε.

### 4.2. Selecting the Hermitian Laplacian

A key aspect of the ICGFT algorithm is selecting an appropriate Laplacian (or adjacency) matrix. This is a classical problem in graph signal processing. One option could be to estimate $Q_{ss}$ from the set of available original graph signals (see for example [17,18,28,29,30,31,41,42]), and then using it as the Hermitian Laplacian. In such a case, we have seen in Section 3 that the precision matrix thus estimated is preserved in the surrogates. Other alternatives make a direct estimation of the Laplacian matrix by minimizing cost functions, which include some regularization constraints [15,16]. However, in a scenario of scarcity, it would be difficult to obtain stable estimates of the Hermitian Laplacian matrix by all these methods.

A different option is to define connections from a general or specific desired connectivity that should be preserved in the surrogates. For example, a general option could be to connect every node only with its neighbors (this is done in [11] in the space domain), so that local properties of the graph signal can be retained in the surrogates. More specific connections can be established depending on the context and the application domain.

A question remains regarding the sign of $w_{nm}^{I}$. Remember that $w_{nm}^{R}=w_{mn}^{R}\geq 0$, but $w_{nm}^{I}=-w_{mn}^{I}$, for example, we may connect two samples because they are neighbors, but this can be made with both a positive or negative value of the imaginary part $w_{nm}^{I}$. As indicated in Equation (2), a different sign selection of $w_{nm}^{I}$ leads to a different smoothness that eventually could be negative. We propose to select the signs in such a way that S(s) is maximized, i.e., by fitting $sign\left[w_{nm}^{I}\right]=sign\left[sin\left(∡s_{m}-∡s_{n}\right)\right]$. We show in Appendix B, that the spectrum bandwidth $B=\lambda_{\max}-\lambda_{\min}$ of the Hermitian Laplacian matrix is lower bounded by

(18) $$B\geq \max\left(l_{\max},\;\frac{S\left(s\right)}{s^{H}s}\right)-l_{\min},$$

where $l_{\max}$ and $l_{\min}$ are respectively the maximum and minimum of the main diagonal elements of the Hermitian Laplacian matrix (which depends only on $w_{nm}^{R}$). Then, given $w_{nm}^{R},\;w_{nm}^{I}$, and s, the lower bound is maximized when S(s) is the maximum. As the key step of surrogating is phase randomization in the transformed domain, it is appropriate to have available the maximum possible bandwidth.

Finally, notice that FT (more precisely its numerical implementation by the discrete Fourier transform (DFT)) can be interpreted as a particular form of selecting the Hermitian Laplacian matrix. This is because it is well-known that the DFT basis vectors are eigenvectors of circulant matrices [43]. So, implicitly, the use of the DFT imposes constraints in the elements of the adjacency matrix. In particular, pairwise connectivity is the same for nodes located at the same “distance” in the node numbering, which implies that stationarity (from the conventional concept of translation) is assumed. Moreover, the required cyclic symmetry of the coefficients imposes additional constraints which may be nonsense in a general context. Also, notice that the eigenvectors of a circulant Hermitian Laplacian matrix do not depend on the specific value of the weights. Hence, much more flexibility can be obtained by defining specific connectivity in concordance with the desired properties of the original graph signal that are to be preserved in the surrogates.

## 5. Experiments

Many applications of signal processing are characterized by a scarcity of signals during the development phase. This, in turn, produces a shortage of instances (vectors of features extracted from the preprocessed signals) for automatic training of classifiers. For example, in automatic non-destructive testing of materials, a classifier is to be trained for every class of defect. This requires a collection of a large number of signals including all the possible defect types, which can be impractical or even impossible [44,45]. Another totally different example is credit card fraud detection [46], where the number of legitimate operations is an order of magnitudes larger than the fraudulent operations. This also happens in multi-class problems where some classes may have a reduced training set [47]. A solution to alleviate scarcity is to generate synthetic replicas using traditional methods (see for example [48] and references therein). But traditional methods are model-oriented, e.g., the model fits some first-order probability density and correlation properties. Model-oriented methods require the definition of an appropriate model and the estimation of the model parameters. But in a scenario of scarcity, both things are rather limited. In contrast, surrogating may be considered a signal-oriented method of synthesis, it has actually already been proposed as a general method for synthesizing multivariate data [38]. Hence, in this experimental section, we consider the application of surrogates to increase the data set for classifier learning. Moreover, notice that to some extent, this is a natural generalization of the use of surrogates in hypothesis testing. In this latter case, the null hypothesis is that the instance under test satisfies a given model (for example a linear model followed by a scalar nonlinearity). But, ultimately, some empirical properties of the original instance (autocorrelation, amplitude distribution, etc) are assumed to represent the model and are accordingly imposed to the surrogates. The same principle can be applied to generate instance replicas for deficient classes in classifier training: The replicas (surrogates) must keep some relevant properties of the original instances. In particular, considering that the instances are graph signals, the surrogates preserve the GSA invariants deduced in Section 3, which relates to preserving the pairwise interrelations defined by the corresponding Hermitian Laplacian matrix.

### 5.1. Automatic Hand Gesture Recognition

In this section, we consider the use of the surrogating methods in a real data case study—Automatic hand gesture recognition. This has been selected due to the complexity of the problem—A large number of classes, with a large and variant number of features. Thus, results should be hopefully extrapolated to simpler cases of automatic classification. Hand gesture recognition is very useful in everyday life for computer–human interaction in applications such as navigating in virtual environments or recognizing hand poses [49]. Originally, hands were tracked by camera images to determine their pose and their 2-D trajectory, but the introduction of depth sensors like Kinect or Leap Motion allowed the ability to obtain 3-D information to recognize the gestures [50]. These applications typically use hidden Markov models (HMM) [51] or support vector machine [52] based classifiers. We have used a public dataset (available at https://github.com/Sasanita/spanish-sign-language-db) composed by 91 approximations of the gestures of Spanish sign language acquired by a Leap Motion sensor. This dataset contains 40 instances per sign. Each instance is a matrix having 42 columns. Every column is a real signal corresponding to the temporal evolution of one observed variable (feature). The whole 42 observed variables describe the trajectory of the fingers and hands and their inclination according to X, Y, and Z axes of a three-dimensional system. The sampling frequency is 30 Hz for all instances, however, the observation time may vary between different instances because the hand sign can be produced at different velocities. Thus, the column dimension of the instance matrices varies from 7 (0.23 s) to 101 (3.36 s) with an average value of 26 (0.86 s). In all cases, the classifier was based on a hidden Markov model (HMM) having seven states. The conditioned probability of the observations to the hidden states was considered a Gaussian mixture of two components. The Viterbi algorithm was used for estimating the most probable sequence of states. Actually, the Hidden Markov Model Toolkit was used (details can be found in [53] and references therein). In all cases the testing of the classifier once trained, was made with original instances not included in the training sets to avoid overfitting.

With the aim of clarifying the experiments, we have defined an Enlarged Training Set (ETS)—a set formed by only a reduced number of original instances enlarged by a varying number of surrogates obtained from them. We also define an Original Training Set (OTS) which is only formed by original instances. We have considered four different types of training sets:

- OTS, the original training set formed by only original instances.
- ETS-ICGFT, an enlarged training set formed by only a reduced number of original instances enlarged by a varying number of surrogates obtained from them by the ICGFT method (Algorithm 1).
- ETS-IAAFT, an enlarged training set formed by only a reduced number of original instances (real part) enlarged by a varying number of surrogates obtained from them by the IAAFT method.
- ETS-*n*IGFT, an enlarged training set formed by only a reduced number of the original instances (real part) enlarged by a varying number of surrogates obtained from them by the method proposed in [11], which uses the (real) GFT. This method does not iterate as no corrections are made in the original graph domain, so we call it non-Iterative GFT (*n*IGFT).

Several details are relevant about the implementation. Thus, in IAAFT the discrete Fourier transform (DFT) was computed having the same size as the transformed column so that the original domain dimension can be preserved once the inverse DFT is computed. On the other hand, ICGFT is conceived to work with complex graph signals, so we first compute the analytic signal corresponding to each one of the 42 columns of the instance matrix (this is a resource that we can always resort to in applying ICGFT to real instances, thus benefiting from the unconstrained phase randomization of the CGFT in contrast with the limited sign randomization of the GFT). The analytic signal is a complex signal, whose real part is the original real signal and whose imaginary part is the Hilbert transform of the original real signal. It can be computed by using a Hilbert transform filter or by Fourier transforming the original real signal, retaining only the spectral content corresponding to the positive frequencies and inverse Fourier transforming. Hence, the analytic signal keeps the information of the original real signal (whose Fourier transform is symmetric), while its complex nature allows the application of ICGFT. Then the graph signal is built by assigning every complex component of the corresponding instance column to the node of a graph. Regarding the Laplacian matrix required in Algorithm 1, it was computed from an adjacency matrix defined as

(19) $$W\left(n,m\right)=\;\left\{ \begin{array}{l} 1\pm j\;\left|n-m\right|=1\\ 0\;otherwise\; \end{array}\right.=\text{​}W^{*}\left(n,m\right)\;\overset{}{\underset{,}{}}$$

where the signs of the imaginary part have been selected as indicated in Section 4.2 (the real part of Equation (19) is retained to implement *n*IGFT). This is made in an effort to replicate the local behavior of the original graph signals (neighbors are connected), but many other options are possible. In particular, we could try to replicate some special properties of the original instances which are known to be relevant for the final goal of classification. This normally should be made with the collaboration of an application domain specialist. Moreover, notice that the selection of normalized weights of magnitude equal 1 in Equation (19) implicitly indicates that no preference is given to the connection of some neighbor samples with respect to other neighbor samples. Actually, the magnitude of the weights connecting neighbor samples is an additional degree of freedom that could be exploited to better replicate in the surrogates some possible nonstationarity of the original signals (e.g., some neighbor samples are much more correlated than others). This requires a detailed analysis of the original signals dynamics, and a non-obvious procedure to deduce the weight magnitudes for all the graph connections. We have preferred instead to keep the simplicity and the general applicability of Equation (19), and to recover the signal dynamics by an “ad hoc” correction step as explained in the following.

In hand gesture recognition, the dynamics of the signals must be captured by the classifier. This is done by the HMM by first determining the successive states through which the signals are passing. Recovering the empirical distribution of the original graph signals may seriously affect the signal dynamics, so we have considered in this case a different type of correction, namely, recovering the original instantaneous frequency, which is a largely accepted measure of the dynamic of complex signals [54]. In such a way we take advantage of working in the complex domain and the “waveform” evolution of the original graph signals and the surrogates will be much more comparable. This specific correction can be expressed in the form

(20) $$s^{\left(i\right)}=C^{\left(i\right)}{\hat{s}}^{\left(i\right)}\;i=1\ldots I,$$

where $C^{\left(i\right)}$ is a diagonal matrix having elements $C^{\left(i\right)}\left(n,n\right)=\exp\left(j\left(∡s_{n}-∡{\hat{s}}_{n}^{\left(i\right)}\right)\right)$.

Two experiments have been conceived to illustrate the interest of using surrogates in two representative scenarios. The first one will be that of overall scarcity: We will assume that only a reduced number of original instances are available in every class, then the instance set will be enlarged by surrogates to improve the quality of the training. In the second scenario, we will consider the imbalanced situation in which there is a significantly smaller number of available instances in some classes than in others.

### 5.2. Experiment 1. Enlarging the Original Training Set: Overall Scarcity

**Figure 1:**
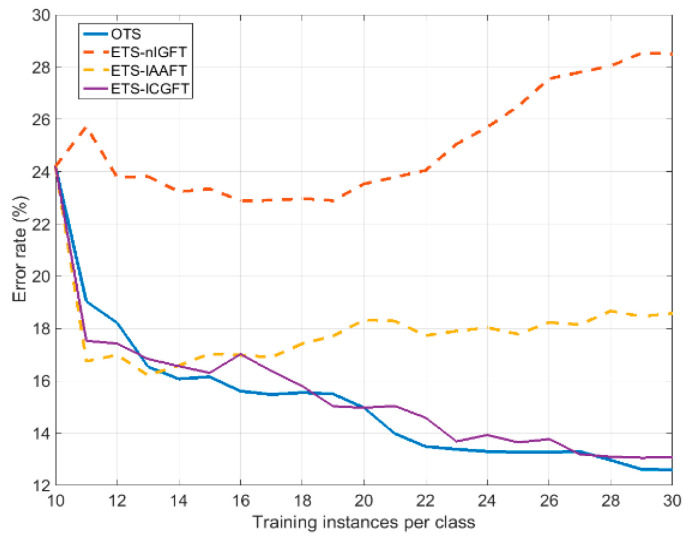
The classification error rate for increasing the number of training instances.

In this experiment, we consider that only a reduced number of instances are available for training in every class, specifically 10. Then this reduced set is enlarged by adding surrogate instances of it to from an ETS. Figure 1 shows the mean error rate achieved by the different surrogating methods as a function of the ETS size, from 10 (only the original 10 training instances per class) to 30 (10 original training instances plus 20 surrogates per class). The mean error rate was evaluated over 10 original instances, which are different from the ones available from training. The error rates of four partitions were averaged. In every partition, the surrogates were generated from a reduced set of 10 original instances to reproduce the practical case of scarcity. We have also included in Figure 1, for comparison, the curves corresponding to an OTS having the same size as the corresponding ETS.

We can see in Figure 1 that training with ETS-ICGFT yields similar results to training with OTS, so that in case of scarcity we can add surrogates keeping the training quality. This is not the same when training with ETS-IAAFT, because, after an initial decrease, the error rate increases with the number of added surrogates. Something similar happens by training with ETS-*n*IGFT, but with much larger error rates. This suggests that ICGFT is able to implement more realistic surrogates and a better oversampling of the instance space. Table 2 compares the error rates corresponding to the use of an ETS of the maximum size (10 original + 20 surrogates) for every class, obtained from different surrogating methods. We also include the error rates corresponding to OTS of size 10 and OTS of size 30.

### 5.3. Experiment 2. Enlarging the Original Training Set: Imbalanced Classes

**Figure 2:**
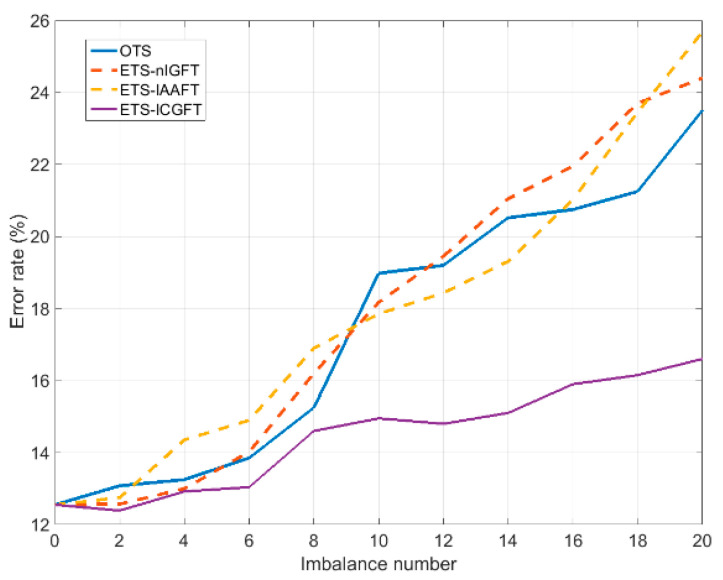
The classification error rate for increasing the imbalance number.

In this experiment, we assume that the number of original training instances is different for every class. Thus, we will consider that 30 original training instances are available for half the classes, while only a number N≤30 is available for the other half (the same for all of them). Figure 2 shows the results corresponding to this experiment: Mean error rate (4 partitions) as a function of the imbalance number. This latter is defined as the difference 30−N, where N varies from 30 (no imbalance) to 10 (largest imbalance). In Figure 2 we have represented the curve corresponding to training with just the original (imbalanced) training set (OTS), as expected the error rate (evaluated again on a set of 10 original instances different from the ones available from training) significantly increases with the imbalance number. Then we show in Figure 2 the error rates obtained by enlarging the deficient class training sets with 30−N surrogate instances, so that all classes have 30 instances for training.

We can see in Figure 2 that only ETS-ICGFT is able to significantly reduce the error corresponding to training with OTS, thus alleviating the problem of imbalanced training. Table 3 shows the error rate averaged over the imbalance number domain for the OTS and for the ETS obtained from the different surrogating methods.

## 6. Conclusions

We have presented a new algorithm for generating surrogates. By considering that instances of any arbitrary domain are defined on the vertices of a graph, much more flexibility can be obtained by the new algorithms in comparison with traditional surrogating methods based on the FT. This flexibility emanates from the definition of the graph topology by the Laplacian matrix. We have considered the general case of surrogating complex graph signals, so a Hermitian Laplacian matrix and an associated CGFT have been defined. Some theoretical analysis and interpretations regarding these new definitions have been made accordingly. This may have a general interest in the new area of GSP. Moreover, by means of the concept of GSA invariants, we have deduced significant properties of the original graph signals that are preserved into the surrogates. 

Experiments on automatic hand gesture recognition have confirmed the efficacy of enlarging the original training set with surrogates and that the best results correspond to the use of ICGFT.

## Figures and Tables

**Table 1:** Graph Specrum Amplitude (GSA) invariants.

Invariant	Equation
Signal energy	$\sum_{n=1}^{N}{\left|s_{n}\right|}^{2}=\sum_{n=1}^{N}{\left|{\hat{s}}_{n}\right|}^{2}$
Frobenious norm of the sample correlation matrix	$\sum_{n=1}^{N}\sum_{m=1}^{N}{\left|s_{n}s_{m}^{*}\right|}^{2}=\sum_{n=1}^{N}\sum_{m=1}^{N}{\left|{\hat{s}}_{n}{\hat{s}}_{m}^{*}\right|}^{2}$
Traces of the sample correlation matrix powers	$trace\left({\left(ss^{H}\right)}^{k}\right)=trace\left({\left(\hat{s}{\hat{s}}^{H}\right)}^{k}\right)$
Smoothness	$s^{H}Ls={\hat{s}}^{H}L\hat{s}$
Precision matrix	If $L=Q_{ss}$ then $Q_{\hat{s}\hat{s}}=Q_{ss}$
Graph Wide-Sense Stationarity	If $C_{ss}=U\Gamma_{ss}U^{H}$ then $C_{\hat{s}\hat{s}}=C_{ss}$

**Table 2:** Error rates for different original and enlarged training sets.

	OTS10	ETS-*n*IGFT	ETS-IAAFT	ETS-ICGFT	OTS30
*Error rate* (%)	24.1	28.5	18.58	13.07	12.6

**Table 3:** Error rate averaged over the imbalance number for the original and enlarged training sets.

	OTS	ETS-*n*IGFT	ETS-IAAFT	ETS-ICGFT
*Error rate* (%)	17.47	17.91	17.92	14.82

## Appendix A. Derivation of Equation (2)

Let us separate the Hermitian adjacency matrix into its real and imaginary parts: $W=W_{R}+jW_{I}$. It is clear that $W_{R}$ is symmetric with non-negative real elements and $W_{I}$ is antisymmetric with real elements, more specifically

(A1) WR(n,m)=Re[W(n,m)]=Re[W(m,n)]≥0  ,WI(n,m)=Im[W(n,m)]=−Im[W(m,n)].

To ease the notation we define Re[W(n,m)]≡wnmR       Im[W(n,m)]≡wnmI. Let us compute the Hermitian Laplacian quadratic form:

(A2) $$\begin{array}{ll} S\left(s\right) & =s^{H}Ls=s^{H}\left(D-W\right)s\\ & =s^{H}\left(D-W_{R}\right)s-js^{H}W_{I}s \end{array}$$

But

(A3) $$\begin{array}{l} s^{H}\left(D-W_{R}\right)s\\ =\sum_{n=1}^{N}\sum_{m=1}^{N}w_{nm}^{R}\left({\left|s_{n}\right|}^{2}-s_{n}^{*}s_{m}\right)\;\\ \;=\sum_{n=1}^{N}\sum_{m=1}^{N}w_{nm}^{R}{\left|s_{n}-s_{m}\right|}^{2}-\;\sum_{n=1}^{N}\sum_{m=1}^{N}w_{nm}^{R}\left({\left|s_{m}\right|}^{2}-s_{n}s_{m}^{*}\right)\;\\ \;=\sum_{n=1}^{N}\sum_{m=1}^{N}w_{nm}^{R}{\left|s_{n}-s_{m}\right|}^{2}-\;\sum_{n=1}^{N}\sum_{m=1}^{N}w_{mn}^{R}\left({\left|s_{m}\right|}^{2}-s_{m}^{*}s_{n}\right)\;\\ \;=\sum_{n=1}^{N}\sum_{m=1}^{N}w_{nm}^{R}{\left|s_{n}-s_{m}\right|}^{2}-\left(s^{H}\left(D-W_{R}\right)s\right).\; \end{array}$$

where we have made use of the symmetry of $W_{R}$, i.e., $w_{nm}^{R}=w_{mn}^{R}$. From Equation (A3) we conclude that

(A4) $$\begin{array}{l} s^{H}\left(D-W_{R}\right)s\\ =\frac{1}{2}\sum_{n=1}^{N}\sum_{m=1}^{N}w_{nm}^{R}{\left|s_{n}-s_{m}\right|}^{2}\\ =\sum_{n=1}^{N-1}\sum_{m>n}^{N}w_{nm}^{R}{\left|s_{n}-s_{m}\right|}^{2}. \end{array}$$

On the other hand

(A5) sHWIs=∑n=1N∑m=1NwnmI sn∗sm=∑n=1N−1∑m>nN(wnmI sn∗sm+wmnI sm∗sn)=∑n=1N−1∑m>nNwnmI(sn∗sm−sm∗sn)=∑n=1N−1∑m>nNwnmI j2Im [sn∗sm]   .

In the first equality of Equation (A5) we have taken into account that $w_{nn}^{I}=0\;\forall n$, and in the second equality that $w_{nm}^{I}=\;-w_{mn}^{I}\;\forall n\neq m$. So finally we obtain:

(A6) S(s)=sHLs==∑n=1N−1∑m>nNwnmR|sn−sm|2+∑n=1N−1∑m>nN2wnmI Im[sn∗sm]      .

## Appendix B. Derivation of Equation (18)

The Schur–Horn theorem [55] applied to the Hermitian Laplacian states that

(A7) $$\sum_{n=1}^{M}\left(\lambda_{n}-l_{n}\right)\geq 0\;M\leq N.$$

where $\lambda_{n}\text{​​}$ and $l_{n}\;n=1\ldots N$ are, respectively, the eigenvalues and the main diagonal elements of the Hermitian Laplacian sorted in non-increasing order. Considering in Equation (A7) M=1 we obtain

(A8) $$\lambda_{1}\text{​}\geq l_{1}\;,$$

which can be written $\lambda_{\max}\text{​}\geq l_{\max}\;$ On the other hand, for M=N Equation (A7) holds with equality, then we can write:

(A9) $$\lambda_{N}+\sum_{n=1}^{N-1}\lambda_{n}=l_{N}+\sum_{n=1}^{N-1}l_{n}.$$

Hence $\;\sum_{n=1}^{N-1}\left(\lambda_{n}-l_{n}\right)=l_{N}-\lambda_{N}\geq 0\;$ and therefore $\lambda_{N}\leq \;l_{N}$, which can be written $\lambda_{\min}\text{​}\leq l_{\min}\;$.

We know that for any vector s

(A10) $$\lambda_{\max}\geq \frac{S\left(s\right)}{s^{H}s}.$$

Hence, given a vector s we may write a lower bound for the spectrum bandwidth $B=\lambda_{\max}-\lambda_{\min}$

(A11) $$B\geq \max\left(l_{\max},\;\frac{S\left(s\right)}{s^{H}s}\right)-l_{\min}.$$

## Appendix C. List of Acronyms

FT	Fourier Transform
GSP	Graph Signal Processing
GFT	Graph Fourier Transform
CGFT	Complex Graph Fourier Transform
GSA	Graph Spectrum Amplitude
GWSS	Graph Wide-Sense Stationary
AAFT	Amplitude Adjusted Fourier Transform surrogating algorithm
IAAFT	Iterative AAFT surrogating algorithm
ICGFT	Iterative CGFT surrogating algorithm
nIGFT	non-Iterative GFT surrogating algorithm
DFT	Discrete Fourier Transform
HMM	Hidden Markov Models
OTS	Original Training Set
ETS	Enlarged Training Set
GMM	Gaussian Mixture Model
